# Notes and Comments

**Published:** 1893-04

**Authors:** 


### Notes and Comments.[1]

ANY one who entertains a real interest in his profession, with a desire to keep apace with the times, will gladly contribute his mite to the onward movement, and will as gladly welcome all criticism. Criticisms and controversies, when carried on with judgment, are wholesome: they are the door-way to true progress and knowledge.

"To him who works in the right direction all things are possible, and the higher he places the limit of his attainments, the greater his attainments will be."—*Dr. Eugene Talbot.*

THE HERBST METHOD OF TREATING PULPS.—According to some observers, the long-hoped-for day has come when we may conscientiously leave root-canals to look after themselves. We remember hearing, some few years back, of the wonderful properties

[1] The assistant editor solicits contributions for this department,—new methods, new remedies and formulas, or any short practical note which may prove of value to the practitioner or student. Address 1506 Arch Street, Philadelphia.

ascribed to cobalt by Dr. Herbst. But we are also under the impression that somebody analyzed a sample and found that the substance was principally arsenic. Certainly a single application of arsenious acid does not always devitalize the whole pulp, and the Herbst method consists in removing the soft tissue as far as the entrance to the canals, with antiseptic care, and then burnishing a tin or gold floor on the bottom of the pulp-chamber. We publish elsewhere an abstract from a paper written by a well-known practitioner in New York, who apparently, after vigorously opposing the method, has become a convert. His communication met with sharp criticism in the discussion which followed. It has also afforded an opportunity for an editorial protest in one of the journals, the writer of which amusingly says that he is unable to see much difference between the "new method" and the slovenly operations of indolent or ignorant operators everywhere, especially in England and Germany!—*Editorial in British Journal of Dental Science.*

---

Weighted Rubber.—A writer in the *Items of Interest*, requesting information concerning weighted rubber, says, "We would like to know whether weighted rubber is more irritating to the gums than ordinary rubber? A late trying experience suggests the inquiry."

Weighted rubber is more or less irritating to the mucous membrane. It is always the best practice in packing, where this rubber is used, to place a layer of the ordinary rubber next to the cast, and owing to the superiority of the strength of the latter it is better to pack a small portion around the pins of the teeth.

---

Extracting.—The brutal and unprofessional practice of some dentists of extracting roots by cutting through the gum and process with the forceps, to grasp a decayed root, has no excuse, and yet it is a common thing to do when the patient is under the effect of gas. Not infrequently the fifth nerve has been severed and facial paralysis induced by too deep a dip of the forceps in the extraction of a wisdom-tooth. Cutting through the gum and process to extract a root below the free margin of the gum is malpractice, and a person so disposed can recover damages for improper laceration of the mouth.—*Extract from a Dental Essay.*

That is simply catachrestic nonsense. There is no tissue that so easily heals as that of the jaws. It is sometimes absolutely necessary to remove a root that is embedded in the jaw, when there is no way of doing this but by excising the alveolus. Is a surgeon never justifiable in cutting to open an abscess, or to remove a tumor?

What does the writer mean by "the fifth nerve"? Does he imagine that there is danger of wounding the Casserian ganglion in extracting a wisdom-tooth? Let him study his anatomy and see where the dentist would be obliged to go to sever the inferior maxillary nerve, or how he would reach the posterior dental in the upper jaw.—*Dental Practitioner and Advertiser.*

---

Mechanics in Dentistry.—In his paper on "Mechanics in Dentistry," Dr. Bonwill makes the following remarkable assertion:

"I think that it was an unfortunate procedure when Dr. was first placed as a title to our names. It is like a big solitaire diamond on the hands of a man who has to labor, whether in dentistry or the trades; he cannot keep his eyes away from it. Nor can he lose sight of the fact that his hand or finger must be placed so that every one else can see it. I appreciated it as much as any one when by certain laws of the colleges a diploma was given me, which I never looked at until the officer demanded it for registration."

The paper was read before the Odontological Society of Pennsylvania, and published in a late issue of this journal. There are valuable thoughts both in the address and in the discussion which followed its reading. But the author, we think, stood in a false position throughout; in fact, in his very first sentence he says that the subject (mechanics) is an unpalatable one,—that "there is no other word in dental language that is held to-day in such disrepute." This is surely a mistaken idea. We all recognize that mechanics is the basis of our work; but to fulfil in the most perfect manner many of the requirements of our profession, one must be more than artisan; he must be an artist, and must have a broad, liberal, professional education. In fact, dentistry seems to form a connecting-link between the professions, art, and mechanics, and in its highest development exemplifies each. The discussion of the paper is well worth re-reading.

---

Indications Justifying the Extraction of Teeth.—In a recent number of the *Dental Office and Laboratory*, Dr. T. F. Chupein, the editor, writes upon the extraction of teeth in preparing the mouth for an artificial denture. We cannot agree with the doctor in all of his remarks upon the subject. First he says, "Should a solitary central incisor or cuspid remain, these should be extracted before attempting to make the artificial substitutes." That, we think, would be the practice of the majority of dentists; but if the central incisor *and* the cuspid should be the only remaining teeth, would it not be equally good practice to extract them? or, if we find two or three of the incisors remaining, should they not be removed before making the artificial denture? A full denture would be more satisfactory, both as to comfort and appearance, than one made to fit around these teeth.

Again, the author says, "If one molar tooth be good on one side of the mouth, and its fellow on the opposite side be in a very dilapidated condition, every effort should be made to restore this tooth, going even to the extent of crowning the root rather than extract it; for, with two molar teeth by which the denture may be clasped, the substitutes will be of infinitely more service to the wearer; such a condition is applicable to both jaws." Now, there is no one who has a higher appreciation of the natural teeth than the writer; but should such a condition exist in his mouth, or that of one of his patients, he would consider it good practice to remove these teeth, feeling sure that better results could be secured by allowing the plate to extend well around the tuberosities in the superior jaw rather than clasp these teeth. It is impossible, of course, to formulate set rules for all cases, and at times difficult to decide just where to draw the line. Experience and judgment must be our guide in determining the practice to pursue.

